# Machine learning–driven immunophenotypic stratification of mixed connective tissue disease, corroborating the clinical heterogeneity

**DOI:** 10.1093/rheumatology/keae158

**Published:** 2024-03-13

**Authors:** Shinji Izuka, Toshihiko Komai, Takahiro Itamiya, Mineto Ota, Yasuo Nagafuchi, Hirofumi Shoda, Kosuke Matsuki, Kazuhiko Yamamoto, Tomohisa Okamura, Keishi Fujio

**Affiliations:** Department of Allergy and Rheumatology, Graduate School of Medicine, The University of Tokyo, Tokyo, Japan; Department of Allergy and Rheumatology, Graduate School of Medicine, The University of Tokyo, Tokyo, Japan; Department of Allergy and Rheumatology, Graduate School of Medicine, The University of Tokyo, Tokyo, Japan; Department of Functional Genomics and Immunological Diseases, Graduate School of Medicine, The University of Tokyo, Tokyo, Japan; Department of Allergy and Rheumatology, Graduate School of Medicine, The University of Tokyo, Tokyo, Japan; Department of Functional Genomics and Immunological Diseases, Graduate School of Medicine, The University of Tokyo, Tokyo, Japan; Department of Allergy and Rheumatology, Graduate School of Medicine, The University of Tokyo, Tokyo, Japan; Department of Functional Genomics and Immunological Diseases, Graduate School of Medicine, The University of Tokyo, Tokyo, Japan; Department of Allergy and Rheumatology, Graduate School of Medicine, The University of Tokyo, Tokyo, Japan; Research Division, Chugai Pharmaceutical Co., Ltd, Yokohama, Kanagawa, Japan; Laboratory for Autoimmune Diseases, RIKEN Center for Integrative Medical Sciences, Yokohama, Kanagawa, Japan; Department of Allergy and Rheumatology, Graduate School of Medicine, The University of Tokyo, Tokyo, Japan; Department of Functional Genomics and Immunological Diseases, Graduate School of Medicine, The University of Tokyo, Tokyo, Japan; Department of Allergy and Rheumatology, Graduate School of Medicine, The University of Tokyo, Tokyo, Japan

**Keywords:** mixed connective tissue disease, systemic lupus erythematosus, idiopathic inflammatory myopathies, systemic sclerosis, machine learning, immunophenotyping

## Abstract

**Objective:**

The objective of this study was to stratify patients with MCTD, based on their immunophenotype.

**Methods:**

We analysed the immunophenotype and transcriptome of 24 immune cell subsets [from patients with MCTD, SLE, idiopathic inflammatory myopathy (IIM) and SSc] from our functional genome database, ImmuNexUT (https://www.immunexut.org/). MCTD patients were stratified by employing machine-learning models, including Random Forest, trained by immunophenotyping data from SLE, IIM and SSc patients. The transcriptomes were analysed with gene set variation analysis (GSVA), and the clinical features of the MCTD subgroups were compared.

**Results:**

This study included 215 patients, including 22 patients with MCTD. Machine-learning models, constructed to classify SLE, IIM and SSc patients, based on immunophenotyping, were applied to MCTD patients, resulting in 16 patients being classified as having an SLEimmunophenotype and 6 as having a non-SLE immunophenotype. Among the MCTD patients, patients with the SLE immunophenotype had higher proportions of Th1 cells {2.85% [interquartile range (IQR) 1.54–3.91] *vs* 1.33% (IQR 0.99–1.74) *P* = 0.027} and plasmablasts [6.35% (IQR 4.17–17.49) *vs* 2.00% (IQR 1.20–2.80) *P* = 0.010]. Notably, the number of SLE-related symptoms was higher in patients with the SLE immunophenotype [2.0 (IQR 1.0–2.0) *vs* 1.0 (IQR 1.0–1.0) *P* = 0.038]. Moreover, the GSVA scores of interferon-α and -γ responses were significantly higher in patients with the SLE immunophenotype in central memory CD8^+^ T cells, while hedgehog signalling was higher in patients with the non-SLE immunophenotype, in five-cell subsets.

**Conclusion:**

This study describes the stratification of MCTD patients, based on immunophenotyping, suggesting the presence of distinct immunological processes behind the clinical subtypes of MCTD.

Rheumatology key messages

- SLE-immunophenotype MCTD patients demonstrated clinical manifestations more closely aligned with SLE.
- They demonstrated an elevation of IFN response in central memory CD8^+^ T cells.
- The non-SLE–immunophenotype MCTD patients exhibited higher hedgehog signalling scores in Th1 cells and plasmablasts.

## Introduction

MCTD is an uncommon CTD characterized by the presence of anti-U1 RNP (U1-RNP) antibodies. MCTD shares clinical features with SLE, PM/DM and SSc [1, 2]. The classification of MCTD, UCTD and overlap syndrome has been the subject of debate, as several criteria exist [1, 3, 4]. However, recent identification of an autoantibody specific to MCTD, anti-survival motor neuron (SMN) complex antibody, supports the distinctiveness of MCTD in autoimmune diseases [5]. Given that some patients develop severe complications, such as pulmonary hypertension [6, 7], gaining an understanding of the pathogenesis of MCTD is important for the development of host-based therapies to improve treatment options. Efforts have been made to stratify patients with systemic immune-mediated diseases (SIDs) into subgroups based on molecular patterns [8] or immunophenotyping data [9, 10]; however, data on immunophenotyping and transcriptomics of MCTD are limited.

Recently, we conducted a large-scale gene-expression analysis across a diverse range of immune cell subsets from peripheral blood mononuclear cells (PBMCs), together with whole-genome sequencing analysis on patients with SIDs, the Immune Cell Gene Expression Atlas from the University of Tokyo (ImmuNexUT) [11]. Here, we further analysed the proportion of immune subsets combined with the gene expression data from ImmuNexUT, focusing specifically on the stratification of MCTD patients. As mentioned earlier, the concept of MCTD remains controversial, since some physicians consider MCTD to be an early phase of other CTDs characterized by the presence of U1-RNP antibodies [2, 12, 13]. This ambiguity of disease classification motivated us to refine the patient stratification according to pathogenetic mechanisms. Thus, we utilized machine-learning models to stratify MCTD patients based on their immunophenotype, thereby revealing the underlying heterogeneity of MCTD in both clinical presentation and transcriptomic characteristics.

## Materials and methods

### Study design and participants

We employed our database, ImmuNexUT [11], to analyse the immunophenotype of patients with MCTD, SLE, IIM and SSc who attended the University of Tokyo hospital. Each patient fulfilled the respective diagnostic or classification criteria [14–21]. Peripheral blood and clinical data were also collected. Among patients with IIM, active disease was defined as presenting at disease onset without any prior treatment, and inactive disease was defined as achievement of a complete clinical response for six consecutive months without any detectable disease activity [22, 23]. This study was approved by the Ethics Committee of the University of Tokyo (approval number: G10095), and written informed consent was obtained from all enrolled participants.

The 24 immune cell subsets that we sorted by flow cytometry were as follows: naïve CD4^+^ T cells (naïve CD4), memory CD4^+^ T cells (Mem CD4), Th1 cells, Th2 cells, Th17 cells, T follicular helper cells (Tfh), fraction I naïve Tregs (Fr. I nTregs), fraction II effector Tregs (Fr. II eTregs), fraction III non-regulatory T cells (Fr. III T), naïve CD8^+^ T cells (naïve CD8), central memory CD8^+^ T cells (CM CD8), effector memory CD8^+^ T cells (EM CD8), CD8^+^ T effector memory CD45RA^+^ cells (TEMRA CD8), NK cells, naïve B cells (naïve B), unswitched memory B cells (USM B), switched memory B cells (SM B), double negative B cells (DN B), plasmablasts (plasmablast), classical monocytes (CL Mono), intermediate monocytes (Int Mono), non-classical monocytes (NC Mono), myeloid dendritic cells (mDC), and plasmacytoid dendritic cells (pDC). The proportions of each subset were calculated; definitions and parent subsets are shown in Supplementary Tables S1 and S2 (available at *Rheumatology* online).

### Machine learning–based stratification of patients

We utilized eight types of supervised machine-learning approaches: Random Forest, Neural Network, eXtreme Gradient Boosting, Support Vector Machines with Linear Kernel, Stabilized Nearest Neighbor Classifier, k-Nearest Neighbors, Gaussian Process, and AdaBoost Classification Trees. Corresponding packages used in R software were rf, nnet, xgbTree, svmLinear, snn, knn, gaussprLinear, and adaboost, respectively. Initially, we partitioned the dataset into training and validation subsets (75:25 ratio). The training subset, comprising 75% of the data, was used to train all the algorithms via the caret package. The objective was to differentiate between diseases (SLE, IIM and SSc) based on the immunophenotyping of 24 immune cell subsets. The remaining 25% of the data served as the validation set to assess the performance of each model. Subsequently, these classifiers were applied to the immunophenotyping data from the patients with MCTD. Collectively, all MCTD patients were stratified into the SLE immunophenotype, the IIM immunophenotype and the SSc immunophenotype. This classification was based on the most frequently predicted outcome across the eight machine-learning models employed. IIM-immunophenotype and SSc-immunophenotype MCTD patients were defined as having a non-SLE immunophenotype.

To investigate the variations in symptoms among the patients with MCTD, we categorized their symptoms into those related to SLE, IIM and SSc. This categorization was adapted from the diagnostic criteria for MCTD, which encompass manifestations resembling SLE, PM/DM or SSc [1]. The SLE components were defined as polyarthritis, lymphadenopathy, malar rash, pericarditis or pleuritis, and leukopenia (≤4000/μl) or thrombocytopenia (≤100 000/μl). The IIM components were defined as muscle weakness, elevated levels of myogenic enzymes, and myogenic abnormalities on electromyograms. The SSc components were defined as sclerodactyly, interstitial lung disease, and oesophageal dysmotility or dilatation.

### Cell and plasma isolation and flow cytometry

PBMCs were isolated immediately after blood was drawn using density gradient separation with Ficoll-Paque (GE healthcare, USA). Erythrocytes were lysed with a potassium ammonium chloride buffer, and non-specific binding was blocked with Fc-gamma receptor antibodies. The PBMCs were sorted into 24 immune cell types using a 14-colour cell sorter, BD FACSAria Fusion (BD Biosciences, USA). The proportions of the 24 immune cell subsets were identified through manual gating using FlowJo Analysis Software (TreeStar, San Jose, CA, USA). Details regarding the antibodies used and the gating strategies used to identify cell types by flow cytometry were described in our previous study [11] (Supplementary Table S1, available at *Rheumatology* online).

### RNA-sequencing

We obtained RNA-sequencing data of 24 immune cell subsets from 20 MCTD patients out of 22 patients and analysed these cases. Genes with low expression [<10 counts or <1 count per million (CPM) in >90% of samples] were excluded. This was followed by a Trimmed Mean of M values (TMM) normalization using the edgeR (v4.3) package in R (v4.1.0) for each cell type. We conducted differentially expressed gene (DEG) analysis using edgeR. The normalized expression data were converted to log-transformed counts per million [log (CPM + 1)]. Batch effects were corrected using ComBat software. Subsequently, gene set variation analysis (GSVA) was conducted and scores were calculated using GSVA software [24]. Gene sets were selected from the Molecular Signature Database (MsigDB) hallmarks collection, which included hedgehog signalling, notch signalling, angiogenesis, Wnt/β catenin signalling, TGF-β signalling, epithelial mesenchymal transition, oxidative phosphorylation, IL-6 Janus kinase/signal transducer and activator of transcription (JAK-STAT) 3 signalling, TNF A signalling via nuclear factor-kappa B (NF-κB), IFN-α response, and IFN-γ response [25].

### Statistical analysis

We conducted a comparison of immune cell proportions and clinical parameters among patients with MCTD, SLE, IIM and SSc using the chi-squared, Kruskal–Wallis and Dunn’s multiple comparisons tests. The parameters, such as immune cell proportions and clinical parameters, and GSVA scores within the stratified MCTD patients were analysed using the chi-squared test or the Wilcoxon rank-sum test, as appropriate. We also conducted multiple logistic regression analyses on the number of SLE components, to control for potential confounding variables, including age, sex, and the dosage of prednisolone. Analyses were performed and figures were generated using R version 4.1.0 and GraphPad Prism version 10.0.2, respectively.

## Results

### Patient clinical features and immunophenotypes

**Figure 1.**
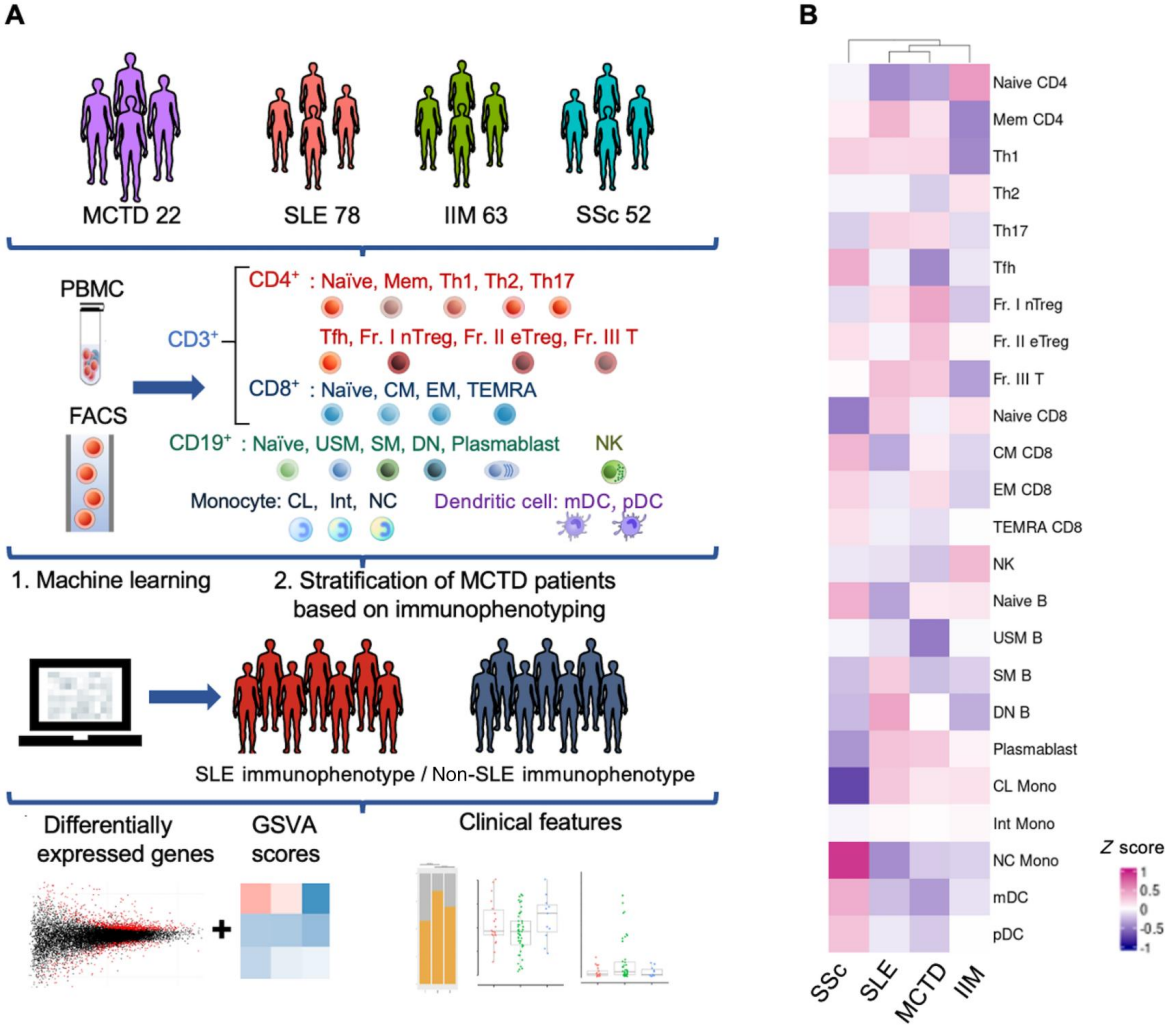
Immunophenotyping of patients with MCTD, SLE, IIM and SSc. (A) Workflow of overall study design. Patients with MCTD were stratified based on immunophenotyping, applying machine learning models that were trained by immunophenotyping of patients with SLE, IIM and SSc. Among the patients with MCTD, the transcriptome and clinical features of those patients with SLE immunophenotype and those with non-SLE immunophenotype were analysed separately. (B) Heatmap showing the mean cell proportions of immunophenotyping of MCTD, SLE, IIM, and SSc. Cell proportions of each disease were hierarchical clustered using the Wards method. GSVA: Gene Set Variation Analysis; IIM: idiopathic inflammatory myopathy

PBMCs and clinical data were collected from a total of 215 patients, including 22 patients with MCTD, 78 with SLE, 63 with IIM and 52 with SSc (Fig. 1A). Regarding the disease manifestations of patients with MCTD, the proportions of SLE components (polyarthritis, lymphadenopathy, malar rash, pericarditis or pleuritis, and leukopenia or thrombocytopenia), IIM components (muscle weakness, elevated levels of myogenic enzymes, and myogenic abnormalities on electromyograms) and SSc components (sclerodactyly, interstitial lung disease, and oesophageal dysmotility or dilatation) were 72.7% (16/22), 36.4% (8/22) and 90.9% (20/22), respectively. This indicates that most MCTD patients had SSc-like symptoms, and a small proportion of patients had IIM-like symptoms.

The percentages of patients who were treatment-naïve were 13.6% (3/22) for MCTD, 12.8% (10/78) for SLE, 63.5% (40/63) for IIM and 67.3% (35/52) for SSc. Among the SLE patients, 17.9% (14/78) of patients received CYC as part of their treatment regimens. Only one patient with IIM was treated with CYC; one other patient with IIM was treated with rituximab. Tocilizumab was administered to 7.7% (4/52) of SSc patients. No patients were administered IVIG. Among the SLE patients, the median SLE disease activity index 2000 (SLEDAI-2K) was 4.00 [interquartile range (IQR) 2.00–9.75], and the median SLE DAS (SLE-DAS) was 4.10 (IQR 1.12–11.30). Among the IIM patients, the median serum creatine kinase levels were 97.5 IU/l (IQR 64.8–224.3), and 55.6% (35/63) of patients were in active disease states. Among the SSc patients, the median modified Rodnan skin score (mTSS) was 4.0 (IQR 2.0–7.0), the median percentage of forced vital capacity (%FVC) was 95.0% (IQR 76.0–104.0) and the median percentage of diffusing capacity for carbon monoxide (%DLCO) was 83.5% (IQR 69.5–96.8). Further characteristics of the four diseases are shown in Supplementary Table S3, available at *Rheumatology* online. We next investigated the similarity of the immunophenotyping among the four diseases. Hierarchical clustering of the four diseases and their immunophenotyping data showed that MCTD had greater similarity with SLE than with other diseases, whereas the immunophenotyping of SSc patients resembled that of patients with other diseases less (Fig. 1B, Supplementary Fig. S1, available at *Rheumatology* online).

### Immunophenotyping-based stratification using machine-learning models

**Figure 2.**
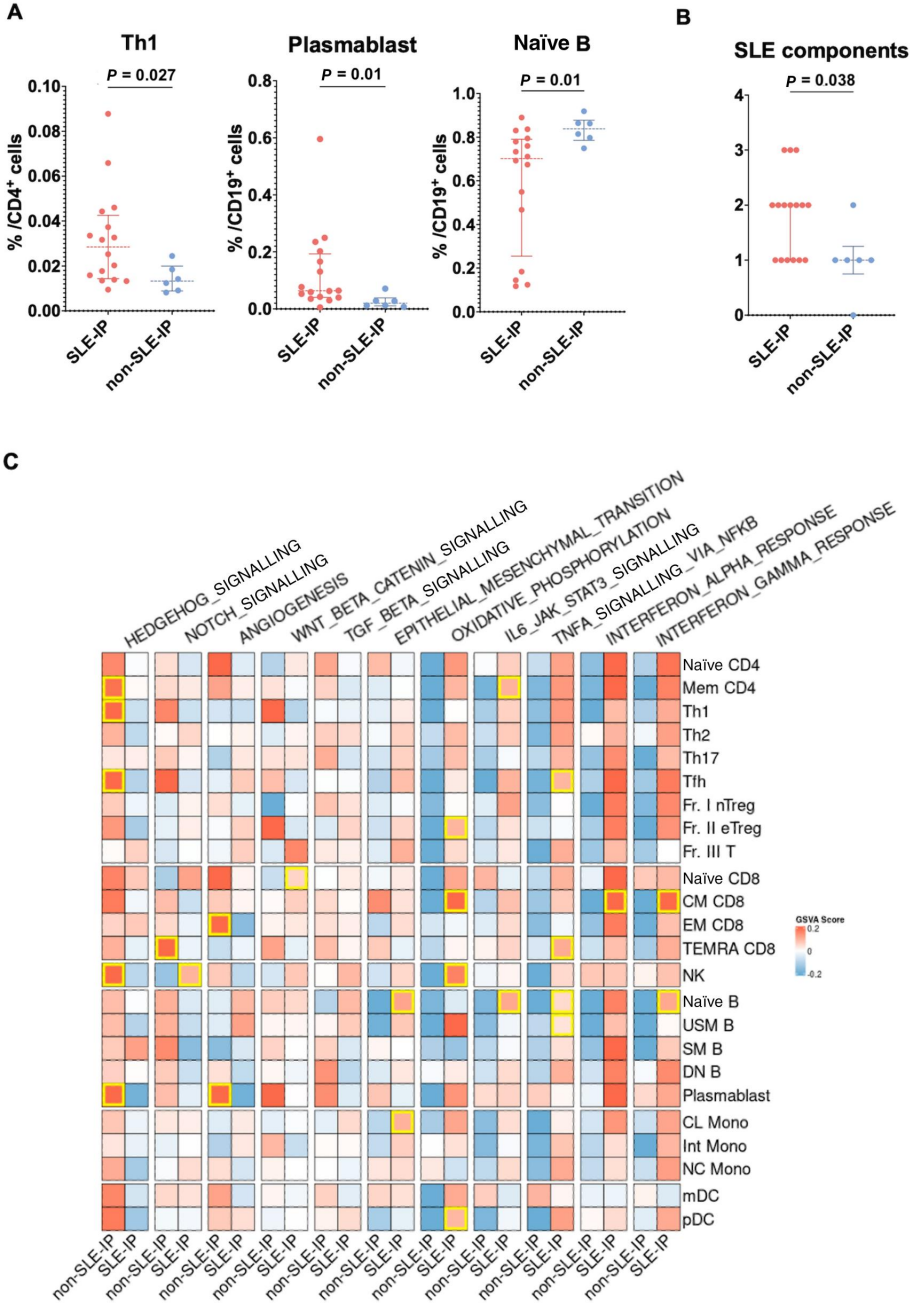
Stratification of MCTD patients. (A) Scatter plots comparing cell proportion data of Th1, plasmablast, and naïve B between MCTD patients with an SLE immunophenotype (*n* = 16) and a non-SLE immunophenotype (*n* = 6). (B). Scatter plots showing the comparison of number of SLE components between MCTD patients with an SLE immunophenotype (*n = *16) and those with a non-SLE immunophenotype (*n = *6). (C) The median GSVA scores of MCTD patients with an SLE immunophenotype (*n = *14) and of those with a non-SLE immunophenotype (*n = *6). Areas marked with yellow squares indicate statistically significant differences, as determined by the Wilcoxon rank-sum test. The Wilcoxon rank-sum test was used to compare two groups. Each point represents an individual measurement. The solid horizontal line within each scatter denotes the median value, while the top and bottom edges of the error bars represent the 75th and 25th percentiles, respectively, indicating the IQR. SLE-IP: SLE immunophenotype; non-SLE-IP: non-SLE immunophenotype; IQR: interquartile range; naïve B: naïve B cell; FDR: false discovery rate; DEGs: differentially expressed genes; TEMRA CD8: CD8^+^ T effector memory CD45RA^+^ cells; GSVA: Gene Set Variation Analysis

We posited that the complex heterogeneity of MCTD might be elucidated through patient stratification based on immunophenotyping, because recent advancements in immunophenotyping-based stratification have contributed to understanding the pathogenesis of other autoimmune diseases [9, 10]. Therefore, we developed machine-learning models for stratifying patients with MCTD based on their immunophenotyping data. Recognizing that MCTD shares certain characteristics with other established diseases such as SLE, IIM and SSc, the models were trained using immunophenotyping data for SLE, IIM and SSc. After the model construction, we applied these models to the data for the MCTD patients (Supplementary Table S4, available at *Rheumatology* online). Of the 22 patients studied, only patient number 11 and patient number 20 exhibited discrepancies in their predicted phenotypes across the eight machine-learning models employed. For these two patients, the inconsistencies were observed in just one of the eight models, suggesting a high level of concordance among the various predictive algorithms (Supplementary Table S5, available at *Rheumatology* online). Notably, of the 22 patients with MCTD, 72.7% (16/22) were classified as having an SLE immunophenotype, and 27.3% (6/22) were classified as having a non-SLE immunophenotype (4/6 IIM immunophenotype and 2/6 SSc immunophenotype) (Supplementary Table S5, available at *Rheumatology* online). These observations, in which a large number of patients were classified as having the SLE immunophenotype, align with the similarity in cell proportions observed between MCTD and SLE (Fig. 1B). Among the MCTD patients, those with an SLE immunophenotype had significantly higher proportions of Th1 cells [2.85% (IQR 1.54–3.91) *vs* 1.33% (IQR 0.99–1.74) *P* = 0.027] and plasmablasts [6.35% (IQR 4.17–17.49) *vs* 2.00% (IQR 1.20–2.80) *P* = 0.010], while the proportion of naïve B in the non-SLE-immunophenotype patients was significantly higher [0.70% (IQR 0.40–0.78) *vs* 0.84% (IQR 0.80–0.86), *P* = 0.010] (Fig. 2A and Supplementary Table S6, available at *Rheumatology* online).

### Clinical features of MCTD patients with the SLE immunophenotype and non-SLE immunophenotype

To identify the differences in the disease manifestations among the stratified MCTD patients, we compared the clinical features. Of note, the number of SLE components was significantly higher in MCTD patients with the SLE immunophenotype [2.0 (IQR 1.0–2.0) *vs* 1.0 (IQR 1.0–1.0) *P* = 0.038] (Fig. 2B and Table 1). This finding may suggest that a clinically meaningful stratification was achieved from the stratification based on the immunophenotypes. Further, multiple logistic regression analyses revealed that the number of SLE components was significantly associated with this immunophenotyping-based stratification (non-SLE immunophenotype *vs* SLE immunophenotype), with an odds ratio of 14.2 and a 95% CI ranging from 1.8 to 389.3, *P* = 0.041. In contrast, no significant differences between the SLE immunophenotype and the non-SLE immunophenotype were observed in relation to the components of IIM or SSc.

**Table 1.** Clinical features of stratified MCTD patients

	SLE immunophenotype (*n* = 16)	Non-SLE immunophenotype (*n* = 6)	*P* value
**Male sex (%)**	2 (12.5)	2 (33.3)	0.29
**Age, years, median [IQR]**	49.50 [41.25, 61.00]	60.0 [46.0, 72.5]	0.32
**Number of SLE components, median [IQR]**	2.0 [1.0, 2.0]	1.0 [1.0, 1.0]	0.038
**Number of IIM components, median [IQR]**	0.0 [0.0, 0.0]	0.0 [0.0, 0.0]	0.39
**Number of SSc components, median [IQR]**	2.0 [1.0, 2.0]	1.0 [0.3, 1.8]	0.33
**Disease duration, years, median [IQR]**	20.0 [13.3, 22.3]	15.5 [11.3, 17.5]	0.58
**Clinical manifestations**			
**RP, (%)**	16 (100.0)	6 (100.0)	>0.99
**Puffy finger, (%)**	15 (93.8)	6 (100.0)	>0.99
**Aseptic meningitis, (%)**	2 (12.5)	0 (0.0)	>0.99
**SLE components**			
**Polyarthritis, (%)**	10 (62.5)	4 (66.7)	0.63
**Lymphadenopathy, (%)**	6 (37.5)	0 (0.0)	0.14
**Malar rash, (%)**	4 (25.0)	1 (16.7)	>0.99
**Pericarditis or pleuritis, (%)**	3 (18.8)	0 (0.0)	0.53
**Leukopenia, (%)**	4 (25.0)	0 (0.0)	0.54
**Thrombocytopenia, (%)**	3 (18.8)	1 (16.7)	>0.99
**IIM components**			
**Proximal muscle weakness, (%)**	1 (6.3)	1 (0.0)	>0.99
**Elevated myogenic enzymes, (%)**	3 (18.8)	1 (16.7)	>0.99
**Pulmonary hypertension, (%)**	4 (25.0)		>0.99
**SSc components, (%)**			
**Sclerodactyly**	3 (18.8)	3 (50.0)	>0.99
**Interstitial lung disease, (%)**	9 (56.3)	2 (33.3)	0.64
**Oesophageal dysmotility or dilatation, (%)**	9 (56.3)	3 (50.0)	>0.99
**Treatment**			
**Prednisolone, (%)**	15 (93.8)	4 (66.7)	0.17
**Prednisolone, mg, median [IQR]**	5.0 [4.4, 7.1]	4.5 [1.0, 6.9]	0.53
**Immunosuppressant, (%)**	7 (43.8)	2 (33.3)	>0.99
**CSA, (%)**	3 (18.8)	0 (0.0)	0.53
**Tacrolimus, (%)**	1 (6.3)	1 (16.7)	0.48
**MTX, (%)**	1 (6.3)	1 (16.7)	0.48
**AZA, (%)**	2 (12.5)	0 (0.0)	>0.99

IIM: idiopathic inflammatory myopathy.

### Transcriptomic features of MCTD patients with the SLE immunophenotype and non-SLE immunophenotype

Next, we investigated the transcriptomic features in MCTD patients by conducting DEG analyses. We compared the SLE immunophenotype and the non-SLE immunophenotype among MCTD patients across 24 immune cell subsets, with the adjustment of covariates such as age, sex and treatment. DEGs were only found within TEMRA CD8; *FCN1*, *MPEG1*, *SERPINA1* and *FPR1* were detected as DEGs [false discovery rate (FDR) <0.05] (Supplementary Fig. S2, available at *Rheumatology* online). Despite the identification of a limited number of DEGs across 24 immune cell subsets, we were able to elucidate the difference in signalling pathways between MCTD patients with the SLE immunophenotype and those with the non-SLE immunophenotype by conducting GSVA (Fig. 2C). GSVA revealed that IFN-α and -γ response scores of CM CD8 were higher in patients with the SLE immunophenotype, compared with patients with the non-SLE immunophenotype (*P* = 0.030, and 0.008, respectively). In addition, TNF-α signalling via NF-κB (in Tfh, TEMRA CD8, naïve B and USM B); IL-6 JAK STAT3 signalling (in Mem CD4 and naïve B); and oxidative phosphorylation (in Fr. II eTreg, CM CD8, and pDC) scores were also enriched in patients with the SLE immunophenotype. Hedgehog signalling scores of Mem CD4, Th1, Tfh, NK and plasmablasts were significantly higher in patients with the non-SLE immunophenotype (*P* = 0.041, 0.011, 0.026, 0.033 and 0.017, respectively). In summary, DEGs were found solely in TEMRA CD8. Among the significantly different proportions of immune cell subsets, naïve B demonstrated higher IFN-γ response, TNF-α signalling via NF-κB, and IL-6 JAK STAT3. In contrast, Th1 and plasmablasts showed higher Hedgehog signalling (Fig. 2C).

## Discussion

Through our immunophenotyping-based stratification using machine-learning models, we revealed distinct clinical and transcriptomic features of MCTD. In addition to showing the similarity of the immunophenotyping of MCTD and SLE, MCTD patients with the SLE immunophenotype had a significantly higher number of clinical features of SLE components. MCTD patients with the SLE immunophenotype exhibited increased proportions of Th1 and plasmablasts and decreased proportions of naïve B cells, suggesting that these cells might be key subsets in pathogenesis. In these cell subsets, Hedgehog signalling pathways were found to be enriched in both Th1 cells and plasmablasts, with angiogenesis enriched in plasmablasts of the non-SLE immunophenotype. Conversely, naïve B cells showed enrichment in IFN-γ response, TNF-α signalling via NF-κB, IL-6 JAK-STAT3 signalling, and epithelial mesenchymal transition pathways. This is the first investigation to evaluate the transcriptome of a wide variety of immune cell subsets in MCTD patients, as well as the stratification of patients based on immunophenotyping.

In a prior study that undertook the stratification of SIDs based on both gene expression and DNA methylation, the majority of patients with MCTD were categorized into an IFN cluster [8]. In the current study, which applied immunophenotype-based stratification, we found that the majority of MCTD patients were stratified into the SLE immunophenotype group (Fig. 2A). This group exhibited a trend of heightened IFN signatures across various immune cell subsets, although significantly enriched IFN signalling was observed solely in CM CD8. Additionally, in a related analysis, Carnero-Montoro *et al.* examined DNA methylation patterns in patients with MCTD and other SIDs, including SLE, SS, RA and SSc [26]. This study uncovered that MCTD shared a highly frequent epigenetic signature with SLE (98%) and SS (98%), particularly at IFN-related sites. Conversely, less frequently shared sites were identified with RA (15%) and SSc (20%) [26]. Taken together, MCTD may exhibit pathological features closely related to SLE, yet it is obvious that MCTD also shares certain pathological characteristics with SSc. This underscores the potential importance of patient stratification in providing effective treatment strategies for MCTD.

To date, numerous studies have focused on the unique features of immunophenotyping, especially in SLE. For instance, the plasmablast signature is supposed to be one of the most robust biomarkers of the disease activity of SLE, and Tfh-dominance with a particularly high proportion of plasmablasts is associated with resistance to treatment [10, 27]. Furthermore, Wangriatisak *et al.* reported that patients with SLE exhibited lower proportions of naïve B cells compared with healthy controls [28], a finding that aligns with the characteristics of the MCTD patients with the SLE immunophenotype observed in this study **(**Fig. 2A**)**. Previously, it was found that IFN-γ–enhanced IL-6 production by B cells facilitated germinal centre formation and autoantibody production in a lupus mouse model [29]. Intriguingly, in the present study, naïve B cells exhibited the combination of enriched IL-6 JAK-STAT3 signalling and IFN-γ response (Fig. 2C), potentially explaining the pathogenesis of SLE-immunophenotype MCTD. In CM CD8, the IFN response and oxidative phosphorylation pathways were found to be significantly enriched in this study (Fig. 2C). Previous studies have shown that exposure to type I IFN is associated with mitochondrial dysfunction in CD8^+^ T cells in patients with SLE [30]. Additionally, another study identified mitochondrial dysfunction, particularly in oxidative phosphorylation processes linked to type I IFN signalling in SLE, though this was found in memory B cells, not in CD8^+^ T cells [31]. The connection between IFN signalling and oxidative phosphorylation may contribute to the pathogenesis of the SLE-immunophenotype MCTD.

In contrast, hedgehog signalling was generally enriched among non-SLE-immunophenotype patients. Hedgehog signalling has been associated with disease progression in the later stages of fibrotic diseases such as SSc [32]. Additionally, hedgehog signalling plays a significant role in B cell development and acts as a macrophage chemoattractant to initiate immune responses [33]. These variations within each MCTD subclass may shed light on differences in pathogenesis and suggest more targeted treatment strategies.

We acknowledge several limitations in this study. First, 86.4% of MCTD patients were undertreated, although there was no statistical difference between MCTD patients with the SLE immunophenotype and those with the non-SLE immunophenotype. Also, in the analysis of the transcriptome data, we made adjustments for batches, including the treatment, to make sure the results were accurate and reliable. This may suggest that the difference in immunophenotyping may reflect the pathophysiology, rather than ongoing treatments. Second, the small number of MCTD patients included in this study necessitates cautious interpretation of the results. Furthermore, the small number of DEGs observed in this study can be attributed to the limited number of patients included. A validation cohort would again be preferable for robust interpretation of these results and for clinical usefulness in terms of determining treatment strategy.

Our study showed the effective stratification of MCTD based on immunophenotyping. This approach may help distinguish MCTD patients in terms of their clinical and transcriptomic features. Our findings hold the potential to inform future precision medicine approaches for patients with MCTD.

## Supplementary Material

keae158_Supplementary_Data

## Data Availability

Data are available at the National Bioscience Database Center (NBDC), with the study accession code E-GEAD-397. The additional data that support the findings of this study are available from the corresponding author, T.K., upon request.
