# Supplementary material for: Machine learning–driven immunophenotypic stratification of mixed connective tissue disease, corroborating the clinical heterogeneity
Source: Rheumatology (Oxford). 2024 Mar 13;64(3):1409–16. doi: 10.1093/rheumatology/keae158 (PMC11879315; doi:10.1093/rheumatology/keae158)

**ONLINE SUPPLEMENTARY MATERIALS**

**Supplementary Table S1. Gating strategy.**

| **Subset** | **Target** | **Parent_pattern1** |
| --- | --- | --- |
| **Naive CD4** | CD4_Lymphocyte_CD3_CD4_NCD4 | CD4_Lymphocyte_CD3_CD4 |
| **Mem CD4** | CD4_Lymphocyte_CD3_CD4_MCD4 | CD4_Lymphocyte_CD3_CD4 |
| **Th1** | CD4_Lymphocyte_CD3_CD4_aTreg | CD4_Lymphocyte_CD3_CD4 |
| **Th2** | CD4_Lymphocyte_CD3_CD4_MCD4_Th1 | CD4_Lymphocyte_CD3_CD4 |
| **Th17** | CD4_Lymphocyte_CD3_CD4_MCD4_Th2 | CD4_Lymphocyte_CD3_CD4 |
| **Tfh** | CD4_Lymphocyte_CD3_CD4_MCD4_TH17 | CD4_Lymphocyte_CD3_CD4 |
| **Fr. I nTreg** | CD4_Lymphocyte_CD3_CD4_MCD4_Tfh | CD4_Lymphocyte_CD3_CD4 |
| **Fr. II eTreg** | CD4_Lymphocyte_CD3_CD4_Fra1 | CD4_Lymphocyte_CD3_CD4 |
| **Fr. III T** | CD4_Lymphocyte_CD3_CD4_Fra3 | CD4_Lymphocyte_CD3_CD4 |
| **Naive CD8** | BCD8_Lymphocyte_CD3_CD8_NCD8 | BCD8_Lymphocyte_CD3_CD8 |
| **CM CD8** | BCD8_Lymphocyte_CD3_CD8_CmCD8 | BCD8_Lymphocyte_CD3_CD8 |
| **EM CD8** | BCD8_Lymphocyte_CD3_CD8_EmCD8 | BCD8_Lymphocyte_CD3_CD8 |
| **TEMRA CD8** | BCD8_Lymphocyte_CD3_CD8_EffectorCD8 | BCD8_Lymphocyte_CD3_CD8 |
| **NK** | NK_Lymphocyte_NK | NK_Lymphocyte |
| **Naive B** | BCD8_Lymphocyte_CD19_NaiB | BCD8_Lymphocyte_CD19 |
| **USM B** | BCD8_Lymphocyte_CD19_UnswMB | BCD8_Lymphocyte_CD19 |
| **SM B** | BCD8_Lymphocyte_CD19_PBSwi_SwiMB | BCD8_Lymphocyte_CD19 |
| **DN B** | BCD8_Lymphocyte_CD19_DNB | BCD8_Lymphocyte_CD19 |
| **Plasmablast** | BCD8_Lymphocyte_CD19_PBSwi_PB | BCD8_Lymphocyte_CD19 |
| **CL Mono** | NK_LymphocyteMonocyte_HLADRposi_  nonNK_CD16nMo | NK_LymphocyteMonocyte_  HLADRposi_nonNK |
| **Int Mono** | NK_LymphocyteMonocyte_HLADRposi_  nonNK_Intermediate | NK_LymphocyteMonocyte_  HLADRposi_nonNK |
| **NC Mono** | NK_LymphocyteMonocyte_HLADRposi_  nonNK_NonClassical | NK_LymphocyteMonocyte_  HLADRposi_nonNK |
| **mDC** | NK_LymphocyteMonocyte_HLADRposi_  nonNK_DCs_mDC | NK_LymphocyteMonocyte_  HLADRposi_nonNK |
| **pDC** | NK_LymphocyteMonocyte_HLADRposi_  nonNK_DCs_pDC | NK_LymphocyteMonocyte_  HLADRposi_nonNK |

**Supplementary Table S2. Subset definition.**

| **Subset name** | **Abbreviation** | **Definition** |
| --- | --- | --- |
| **Naive CD4 T cells** | Naive CD4 | CD3+/CD4+CD8-/CCR7+CD45RA+ |
| **Memory CD4 T cells** | Mem CD4 | CD3+/CD4+CD8-/non-naive CD4+/CD25- |
| **T helper 1 cells** | Th1 | CD3+/CD4+CD8-/non-naive CD4+/CD25-/CXCR5-CCR6-/CXCR3+CCR4- |
| **T helper 2 cells** | Th2 | CD3+/CD4+CD8-/non-naive CD4+/CD25-/CXCR5-CCR6-/CXCR3-CCR4+ |
| **T helper 17 cells** | Th17 | CD3+/CD4+CD8-/non-naive CD4+/CD25-/CXCR5-CCR6+/CXCR3- |
| **T follicular helper cells** | Tfh | CD3+/CD4+CD8-/non-naive CD4+/CD25-/CXCR5+ |
| **Fraction I naive regulatory T cells** | Fr. I nTreg | CD3+/CD4+CD8-/CD25+CD45RA+ |
| **Fraction II effector regulatory T cells** | Fr. II eTreg | CD3+/CD4+CD8-/CD25++CD45RA- |
| **Fraction III non-regulatory T cells** | Fr. III T | CD3+/CD4+CD8-/CD25+CD45RA- |
| **Naive CD8 T cells** | Naive CD8 | CD3+CD19-/CD4-CD8+/CD45RA+CCR7+ |
| **Central memory CD8 T cells** | CM CD8 | CD3+CD19-/CD4-CD8+/CD45RA-CCR7+ |
| **Effector memory CD8 T cells** | EM CD8 | CD3+CD19-/CD4-CD8+/CD45RA-CCR7- |
| **CD8 T effector memory CD45RA+ cells** | TEMRA CD8 | CD3+CD19-/CD4-CD8+/CD45RA+CCR7- |
| **Natural killer cells** | NK | CD3-CD19-/CD14-/CD56+ |
| **Naive B cells** | Naive B | CD3-CD19+/IgD+CD27- |
| **Unswitched memory B cells** | USM B | CD3-CD19+/IgD+CD27+ |
| **Switched memory B cells** | SM B | CD3-CD19+/IgD-CD27+/CD38- |
| **Double negative B cells** | DN B | CD3-CD19+/IgD-CD27- |
| **Plasmablasts** | Plasmablast | CD3-CD19+/IgD-CD27++/CD38+ |
| **Classical monocytes** | CL Mono | CD3-CD19-/HLADR+/CD56-/CD14+CD16- |
| **Intermediate monocytes** | Int Mono | CD3-CD19-/HLADR+/CD56-/CD14++CD16+ |
| **Non-classical monocytes** | NC Mono | CD3-CD19-/HLADR+/CD56-/CD14dimCD16+ |
| **Myeloid dendritic cells** | mDC | CD3-CD19-/HLADR+/CD56-/CD14-CD16-/CD11c+CD123- |
| **Plasmacytoid dendritic cells** | pDC | CD3-CD19-/HLADR+/CD56-/CD14-CD16-/CD11c-CD123+ |

**Supplementary Table S3. Clinical features and immunophenotyping of all patients.**

|  | **MCTD (n=22)** | **SLE (n=78)** | **IIM (n=63)** | **SSc (n=52)** | **p value** |
| --- | --- | --- | --- | --- | --- |
| **Male sex (%)** | 4 (18.2) | 10 (12.8) | 17 (27.0) | 3 (5.8) | 0.014 |
| **Age, median [IQR] years** | 50.0 [42.3, 64.8] | 46.5 [36.3, 56.0] | 57.0 [46.5, 69.0] | 67.5 [56.8, 73.3] | <0.001 |
| **Disease duration, median [IQR] years** | 17.5 [11.3, 21.8] | 12.5 [6.0, 20.0] | 3.0 [0.0, 11.5] | 15.5 [7.0, 26.5] | <0.001 |
| **Symptoms and laboratory findings** |  |  |  |  |  |
| **Raynauds (%)** | 22 (100.0) | 5 (6.4) |  |  | <0.001 |
| **Polyarthritis, (%)** | 12 (54.6) | 17 (21.8) | 1 (1.6) | 9 (17.3) | <0.001 |
| **Skin rash (%)** | 5 (22.7) | 19 (24.4) | 50 (79.4) |  | 1 |
| **Pericarditis or pleuritis, (%)** | 3 (13.6) | 20 (25.6) |  |  | 0.389 |
| **Leukopenia (%)** | 4 (18.2) | 23 (29.5) |  |  | 0.416 |
| **Thrombocytopenia (%)** | 4 (18.2) | 4 (5.1) |  |  | 0.002 |
| **Muscle weakness (%)** | 1 (4.6) | 2 (2.6) | 46 (73.02) | 0 ( 0) | <0.001 |
| **CK, median, [IQR] U/mL** | 59.0 [47.0, 177.0] |  | 97.5 [64.8, 224.3] |  | 0.106 |
| **Interstitial lung disease (%)** | 11 (50.0) | 10 (12.8) | 40 (63.5) | 23 (44.2) | <0.001 |
| **Sclerodactyly (%)** | 6 (27.2) | 0 (0) | 0 (0) | 52 (100) | 1 |
| **Pulmonary hypertension (%)** | 5 (22.7) | 2 (2.6) |  | 0 (0) | <0.001 |
| **%Vital capacity, median, [IQR]** | 86.75 [79.5, 102.0] |  |  | 95.0 [76.0, 104.0] | 0.738 |
| **%Diffusing capacity for carbon monoxide, median, [IQR]** | 75.5 [67.8, 96.9] |  |  | 83.5 [69.5, 96.8] | 0.552 |
| **Esophageal dysmotility or dilatation, (%)** | 12 (54.5) | 0 (0) | 0 (0) | 34 (65.4) | 0.298 |
| **C-reactive protein, median, [IQR]** | 0.12 [0.05, 0.34] | 0.09 [0.03, 0.32] | 0.07 [0.04, 0.28] | 0.08 [0.03, 0.14] | 0.411 |
| **Erythrocyte sedimentation rate, median, [IQR]** | 20.0 [13.0, 44.75] | 22.5 [12.0, 42.3] | 18.0 [8.0, 42.0] | 15.0 [11.0, 22.0] | 0.186 |
| **Immunoglobulin G, median, [IQR]** | 1551.5 [1388.5, 1958.8] | 1433.0 [1061.0, 1812.0] | 1316.0 [991.8, 1696.8] | 1452.0 [1118.5, 1680.0] | 0.09 |
| **Creatinine, median, [IQR]** | 0.70 [0.57, 0.77] | 0.67 [0.57, 0.81] | 0.6050 [0.51, 0.78] | 0.63 [0.55, 0.71] | 0.334 |
| **Treatment** |  |  |  |  |  |
| **Dose of prednisolone, median, [IQR]** | 5.0 [4.0, 7.4] | 5.0 [3.1, 7.5] | 5.0 [0.0, 7.5] | 0.0 [0.0, 0.0] | <0.001 |
| **Hydroxychloroquine (%)** | 1 (4.5) | 28 (35.9) | 0 (0) | 0 (0) | <0.001 |
| **Tacrolimus (%)** | 2 (9.1) | 11 (14.1) | 11 (17.5) | 4 (7.7) | 0.45 |
| **Cyclosporine A (%)** | 2 (9.1) | 9 (11.5) | 7 (11.1) | 1 (1.9) | 0.181 |
| **Mycophenolate mofetil (%)** | 0 (0) | 14 (17.9) | 0 (0) | 3 (5.8) | <0.001 |
| **Azathioprine (%)** | 3 (13.6) | 9 (11.5) | 2 (3.2) | 4 (7.7) | 0.05 |
| **Methotrexate (%)** | 2 (9.1) | 3 (3.8) | 9 (14.3) | 4 (7.7) | 0.15 |
| **Salazosulfapyridine (%)** | 0 (0) | 0 (0.0) | 0 (0) | 3 (5.8) | 0.042 |
| **Mizoribine (%)** | 1 (4.5) | 3 (3.8) | 1 (1.6) | 0 (0) | 0.396 |
| **Immunophenotype** |  |  |  |  |  |
| **CD4 T cells:** |  |  |  |  |  |
| **Naive CD4 (median [IQR])** | 0.2955 [0.1998, 0.3763] | 0.2742 [0.1592, 0.3744] | 0.4091 [0.3124, 0.5232] | 0.3374 [0.2412, 0.3929] | <0.001 |
| **Mem CD4 (median [IQR])** | 0.5461 [0.4430, 0.6346] | 0.5641 [0.4620, 0.7069] | 0.4481 [0.3318, 0.5840] | 0.5417 [0.4739, 0.6071] | <0.001 |
| **Th1 (median [IQR])** | 0.0193 [0.0136, 0.0333] | 0.0238 [0.0155, 0.0351] | 0.0138 [0.0084, 0.0197] | 0.0211 [0.0166, 0.0286] | <0.001 |
| **Th2 (median [IQR])** | 0.0243 [0.0191, 0.0381] | 0.0331 [0.0178, 0.0449] | 0.0303 [0.0194, 0.0510] | 0.0317 [0.0229, 0.0453] | 0.236 |
| **Th17 (median [IQR])** | 0.0407 [0.0195, 0.0500] | 0.0399 [0.0235, 0.0683] | 0.0271 [0.0204, 0.0422] | 0.0249 [0.0191, 0.0353] | 0.022 |
| **Tfh (median [IQR])** | 0.0805 [0.0489, 0.1008] | 0.0924 [0.0690, 0.1256] | 0.0893 [0.0701, 0.1245] | 0.1136 [0.0919, 0.1562] | <0.001 |
| **Fr. I nTreg (median [IQR])** | 0.0228 [0.0151, 0.0375] | 0.0203 [0.0129, 0.0300] | 0.0152 [0.0099, 0.0256] | 0.0173 [0.0147, 0.0251] | 0.045 |
| **Fr. II eTreg (median [IQR])** | 0.0101 [0.0081, 0.0167] | 0.0087 [0.0053, 0.0135] | 0.0085 [0.0056, 0.0148] | 0.0097 [0.0069, 0.0149] | 0.215 |
| **Fr. III T (median [IQR])** | 0.0412 [0.0345, 0.0684] | 0.0452 [0.0318, 0.0694] | 0.0340 [0.0234, 0.0504] | 0.0480 [0.0344, 0.0576] | 0.002 |
| **CD8 T cells:** |  |  |  |  |  |
| **Naive CD8 (median [IQR])** | 0.2491 [0.1404, 0.4277] | 0.3700 [0.1338, 0.5373] | 0.2780 [0.1240, 0.4910] | 0.1279 [0.0823, 0.2623] | 0.001 |
| **CM CD8 (median [IQR])** | 0.0232 [0.0128, 0.0512] | 0.0224 [0.0113, 0.0388] | 0.0271 [0.0144, 0.0411] | 0.0432 [0.0305, 0.0556] | <0.001 |
| **EM CD8 (median [IQR])** | 0.1454 [0.0805, 0.2339] | 0.1217 [0.0718, 0.1998] | 0.1145 [0.0702, 0.1827] | 0.1499 [0.1192, 0.2189] | 0.037 |
| **TEMRA CD8 (median [IQR])** | 0.1351 [0.0836, 0.2488] | 0.1592 [0.0618, 0.2295] | 0.1376 [0.0830, 0.2200] | 0.1955 [0.1259, 0.2764] | 0.282 |
| **NK:** |  |  |  |  |  |
| **NK (median [IQR])** | 0.1002 [0.0488, 0.1491] | 0.1034 [0.0497, 0.1603] | 0.1391 [0.0911, 0.2457] | 0.1251 [0.0706, 0.1822] | 0.068 |
| **B cells:** |  |  |  |  |  |
| **Naive B (median [IQR])** | 0.7545 [0.5805, 0.8266] | 0.5870 [0.3715, 0.7756] | 0.7312 [0.5756, 0.8162] | 0.7773 [0.6656, 0.8455] | <0.001 |
| **USM B (median [IQR])** | 0.0275 [0.0188, 0.0449] | 0.0435 [0.0272, 0.0740] | 0.0512 [0.0315, 0.0860] | 0.0505 [0.0311, 0.0767] | <0.001 |
| **SM B (median [IQR])** | 0.0896 [0.0598, 0.1401] | 0.1345 [0.0849, 0.2568] | 0.0964 [0.0582, 0.1700] | 0.0947 [0.0607, 0.1562] | 0.001 |
| **DN B (median [IQR])** | 0.0373 [0.0253, 0.1047] | 0.0612 [0.0316, 0.1227] | 0.0312 [0.0185, 0.0504] | 0.0302 [0.0205, 0.0473] | <0.001 |
| **Plasmablast (median [IQR])** | 0.0558 [0.0286, 0.1173] | 0.0603 [0.0318, 0.1520] | 0.0336 [0.0148, 0.0864] | 0.0175 [0.0105, 0.0292] | <0.001 |
| **Monocytes:** |  |  |  |  |  |
| **CL Mono (median [IQR])** | 0.8538 [0.7891, 0.9008] | 0.8638 [0.8269, 0.8959] | 0.8532 [0.7974, 0.8916] | 0.7747 [0.7374, 0.8136] | <0.001 |
| **Int Mono (median [IQR])** | 0.0409 [0.0282, 0.0535] | 0.0367 [0.0243, 0.0518] | 0.0360 [0.0236, 0.0576] | 0.0429 [0.0302, 0.0544] | 0.003 |
| **NC Mono (median [IQR])** | 0.0429 [0.0257, 0.0626] | 0.0345 [0.0165, 0.0520] | 0.0449 [0.0135, 0.0860] | 0.1082 [0.0805, 0.1404] | <0.001 |
| **Dendritic cells (DC):** |  |  |  |  |  |
| **mDC (median [IQR])** | 0.0056 [0.0042, 0.0119] | 0.0076 [0.0048, 0.0123] | 0.0075 [0.0046, 0.0128] | 0.0114 [0.0087, 0.0190] | <0.001 |
| **pDC (median [IQR])** | 0.0060 [0.0047, 0.0079] | 0.0064 [0.0036, 0.0101] | 0.0066 [0.0043, 0.0104] | 0.0085 [0.0057, 0.0112] | <0.001 |

naïve CD4 = naïve CD4^+^ T cells, Mem CD4 = memory CD4^+^ T cells, Th1 = T helper 1 cells, Th2 = T helper 2 cells, Th17 = T helper 17 cells, Tfh = T follicular helper cells, Fr. I nTreg = fraction I naïve regulatory T cells, Fr. II eTreg = fraction II effector regulatory T cells, Fr. III T = fraction III non-regulatory T cells, naïve CD8 = naïve CD8^+^ T cells, CM CD8 = central Memory CD8^+^ T cells, EM CD8 = effector memory CD8^+^ T cells, TEMRA CD8 = CD8^+^ T effector memory CD45RA^+^ cells, NK = natural killer cells, naïve B = naïve B cells, USM B = unswitched memory B cells, SM B = switched memory B cells, DN B = double negative B cells, plasmablasts = plasmablasts, CL Mono = classical monocytes, Int Mono = intermediate monocytes, NC Mono = non-classical monocytes, mDC = myeloid dendritic cells, pDC = plasmacytoid dendritic cells

**Supplementary Table S4. Performance of machine learning models.**

|  | **Overall Accuracy** | **95% CI** | **No Information Rate** | **P-Value [Acc > NIR]** | **Kappa** | **Mcnemar's Test P-Value** |
| --- | --- | --- | --- | --- | --- | --- |
| **Random Forest** | 0.6383 | 0.4852, 0.7733 | 0.5106 | 0.05358 | 0.442 | 0.37152 |
| **Neural Network** | 0.6596 | 0.5069, 0.7914 | 0.5532 | 0.09241 | 0.4719 | 0.07343 |
| **eXtreme Gradient Boosting** | 0.6383 | 0.4852, 0.7733 | 0.5532 | 0.1522 | 0.4381 | 0.1753 |
| **Support Vector Machines** | 0.617 | 0.4638, 0.7549 | 0.5319 | 0.1531 | 0.4076 | 0.2895 |
| **Stabilized Nearest Neighbor** | 0.6383 | 0.4852, 0.7733 | 0.5319 | 0.0935 | 0.4397 | 0.3496 |
| **k-Nearest Neighbors** | 0.6383 | 0.4852, 0.7733 | 0.5106 | 0.05358 | 0.442 | 0.37152 |
| **Gaussian Process** | 0.6383 | 0.4852, 0.7733 | 0.5106 | 0.05358 | 0.4413 | 0.51526 |
| **AdaBoost Classification Trees** | 0.6596 | 0.5069, 0.7914 | 0.4894 | 0.01392 | 0.4771 | 0.50617 |

**Supplementary Table S5. Machine learning-based stratification of MCTD patients.**

| **Patient number** | **Random Forest** | **Neural Network** | **eXtreme Gradient Boosting** | **Support Vector Machines** | **Stabilized Nearest Neighbor** | **k-Nearest Neighbors** | **Gaussian Process** | **AdaBoost Classification Trees** | **Result** | **Stratification** |
| --- | --- | --- | --- | --- | --- | --- | --- | --- | --- | --- |
| **1** | SLE-IP | SLE-IP | SLE-IP | SLE-IP | SLE-IP | SLE-IP | SLE-IP | SLE-IP | SLE-IP | SLE-IP |
| **2** | SLE-IP | SLE-IP | SLE-IP | SLE-IP | SLE-IP | SLE-IP | SLE-IP | SLE-IP | SLE-IP | SLE-IP |
| **3** | SLE-IP | SLE-IP | SLE-IP | SLE-IP | SLE-IP | SLE-IP | SLE-IP | SLE-IP | SLE-IP | SLE-IP |
| **4** | SLE-IP | SLE-IP | SLE-IP | SLE-IP | SLE-IP | SLE-IP | SLE-IP | SLE-IP | SLE-IP | SLE-IP |
| **5** | IIM-IP | IIM-IP | IIM-IP | IIM-IP | IIM-IP | IIM-IP | IIM-IP | IIM-IP | IIM-IP | non-SLE-IP |
| **6** | SLE-IP | SLE-IP | SLE-IP | SLE-IP | SLE-IP | SLE-IP | SLE-IP | SLE-IP | SLE-IP | SLE-IP |
| **7** | SLE-IP | SLE-IP | SLE-IP | SLE-IP | SLE-IP | SLE-IP | SLE-IP | SLE-IP | SLE-IP | SLE-IP |
| **8** | IIM-IP | IIM-IP | IIM-IP | IIM-IP | IIM-IP | IIM-IP | IIM-IP | IIM-IP | IIM-IP | nonSLE-IP |
| **9** | SLE-IP | SLE-IP | SLE-IP | SLE-IP | SLE-IP | SLE-IP | SLE-IP | SLE-IP | SLE-IP | SLE-IP |
| **10** | SLE-IP | SLE-IP | SLE-IP | SLE-IP | SLE-IP | SLE-IP | SLE-IP | SLE-IP | SLE-IP | SLE-IP |
| **11** | IIM-IP | IIM-IP | IIM-IP | IIM-IP | SLE-IP | IIM-IP | IIM-IP | IIM-IP | IIM-IP | non-SLE-IP |
| **12** | SLE-IP | SLE-IP | SLE-IP | SLE-IP | SLE-IP | SLE-IP | SLE-IP | SLE-IP | SLE-IP | SLE-IP |
| **13** | SSc-IP | SSc-IP | SSc-IP | SSc-IP | SSc-IP | SSc-IP | SSc-IP | SSc-IP | SSc-IP | non-SLE-IP |
| **14** | SSc-IP | SSc-IP | SSc-IP | SSc-IP | SSc-IP | SSc-IP | SSc-IP | SSc-IP | SSc-IP | non-SLE-IP |
| **15** | SLE-IP | SLE-IP | SLE-IP | SLE-IP | SLE-IP | SLE-IP | SLE-IP | SLE-IP | SLE-IP | SLE-IP |
| **16** | SLE-IP | SLE-IP | SLE-IP | SLE-IP | SLE-IP | SLE-IP | SLE-IP | SLE-IP | SLE-IP | SLE-IP |
| **17** | SLE-IP | SLE-IP | SLE-IP | SLE-IP | SLE-IP | SLE-IP | SLE-IP | SLE-IP | SLE-IP | SLE-IP |
| **18** | SLE-IP | SLE-IP | SLE-IP | SLE-IP | SLE-IP | SLE-IP | SLE-IP | SLE-IP | SLE-IP | SLE-IP |
| **19** | SLE-IP | SLE-IP | SLE-IP | SLE-IP | SLE-IP | SLE-IP | SLE-IP | SLE-IP | SLE-IP | SLE-IP |
| **20** | SLE-IP | SLE-IP | SLE-IP | SLE-IP | SLE-IP | SLE-IP | SSc-IP | SLE-IP | SLE-IP | SLE-IP |
| **21** | IIM-IP | IIM-IP | IIM-IP | IIM-IP | IIM-IP | IIM-IP | IIM-IP | IIM-IP | IIM-IP | non-SLE-IP |
| **22** | SLE-IP | SLE-IP | SLE-IP | SLE-IP | SLE-IP | SLE-IP | SLE-IP | SLE-IP | SLE-IP | SLE-IP |

SLE-IP = SLE-immunophenotype MCTD, non-SLE-IP = non-SLE-immunophenotype MCTD.

**Supplementary Table S6. Immunophenotype of stratified MCTD patients.**

|  | **SLE-IP (n=16)** | **non-SLE-IP (n=6)** | **p value** |
| --- | --- | --- | --- |
| **Naive CD4, %, median, IQR** | 0.2955 [0.1785, 0.3392] | 0.2903 [0.2299, 0.4674] | 0.37 |
| **Mem CD4, %, median, IQR** | 0.5461 [0.4823, 0.6467] | 0.4910 [0.3943, 0.5857] | 0.26 |
| **Th1, %, median, IQR** | 0.0285 [0.0154, 0.0391] | 0.0133 [0.0099, 0.0174] | 0.027 |
| **Th2, %, median, IQR** | 0.0226 [0.0184, 0.0374] | 0.0271 [0.0227, 0.0433] | 0.45 |
| **Th17, %, median, IQR** | 0.0424 [0.0233, 0.0499] | 0.0298 [0.0195, 0.0525] | 0.8 |
| **Tfh, %, median, IQR** | 0.0759 [0.0486, 0.0968] | 0.0905 [0.0658, 0.1092] | 0.59 |
| **Fr. I nTreg, %, median, IQR** | 0.0250 [0.0154, 0.0393] | 0.0217 [0.0121, 0.0301] | 0.49 |
| **Fr. II eTreg, %, median, IQR** | 0.0117 [0.0071, 0.0178] | 0.0087 [0.0083, 0.0099] | 0.64 |
| **Fr. III T, %, median, IQR** | 0.0534 [0.0357, 0.0726] | 0.0371 [0.0331, 0.0420] | 0.49 |
| **Naive CD8, %, median, IQR** | 0.2882 [0.1904, 0.4049] | 0.1525 [0.0457, 0.4618] | 0.64 |
| **CM CD8, %, median, IQR** | 0.0223 [0.0125, 0.0320] | 0.0389 [0.0160, 0.0608] | 0.45 |
| **EM CD8, %, median, IQR** | 0.1460 [0.1118, 0.2444] | 0.1106 [0.0564, 0.1551] | 0.45 |
| **TEMRA CD8, %, median, IQR** | 0.1323 [0.0760, 0.2706] | 0.1457 [0.0994, 0.1985] | 0.91 |
| **NK, %, median, IQR** | 0.0672 [0.0458, 0.1297] | 0.1467 [0.1151, 0.2235] | 0.083 |
| **Naive B, %, median, IQR** | 0.7018 [0.3963, 0.7833] | 0.8385 [0.8016, 0.8638] | 0.01 |
| **USM B, %, median, IQR** | 0.0300 [0.0192, 0.0501] | 0.0238 [0.0187, 0.0389] | 0.45 |
| **SM B, %, median, IQR** | 0.1057 [0.0652, 0.1774] | 0.0632 [0.0490, 0.0817] | 0.1 |
| **DN B, %, median, IQR** | 0.0476 [0.0241, 0.1314] | 0.0292 [0.0256, 0.0346] | 0.18 |
| **Plasmablast, %, median, IQR** | 0.0635 [0.0417, 0.1749] | 0.0200 [0.0120, 0.0280] | 0.01 |
| **CL Mono, %, median, IQR** | 0.8633 [0.8022, 0.9025] | 0.8383 [0.7946, 0.8515] | 0.64 |
| **Int Mono, %, median, IQR** | 0.0409 [0.0282, 0.0526] | 0.0391 [0.0272, 0.0591] | 0.8 |
| **NC Mono, %, median, IQR** | 0.0333 [0.0244, 0.0496] | 0.0584 [0.0562, 0.0735] | 0.11 |
| **mDC, %, median, IQR** | 0.0056 [0.0047, 0.0103] | 0.0074 [0.0042, 0.0119] | >0.99 |
| **pDC, %, median, IQR** | 0.0056 [0.0044, 0.0076] | 0.0075 [0.0057, 0.0097] | 0.26 |

naïve CD4 = naïve CD4^+^ T cells, Mem CD4 = memory CD4^+^ T cells, Th1 = T helper 1 cells, Th2 = T helper 2 cells, Th17 = T helper 17 cells, Tfh = T follicular helper cells, Fr. I nTreg = fraction I naïve regulatory T cells, Fr. II eTreg = fraction II effector regulatory T cells, Fr. III T = fraction III non-regulatory T cells, naïve CD8 = naïve CD8^+^ T cells, CM CD8 = central Memory CD8^+^ T cells, EM CD8 = effector memory CD8^+^ T cells, TEMRA CD8 = CD8^+^ T effector memory CD45RA^+^ cells, NK = natural killer cells, naïve B = naïve B cells, USM B = unswitched memory B cells, SM B = switched memory B cells, DN B = double negative B cells, plasmablasts = plasmablasts, CL Mono = classical monocytes, Int Mono = intermediate monocytes, NC Mono = non-classical monocytes, mDC = myeloid dendritic cells, pDC = plasmacytoid dendritic cells

**Supplemental Figure S1.** **Cell proportions in patients with MCTD, SLE, IIM, and SSc.**


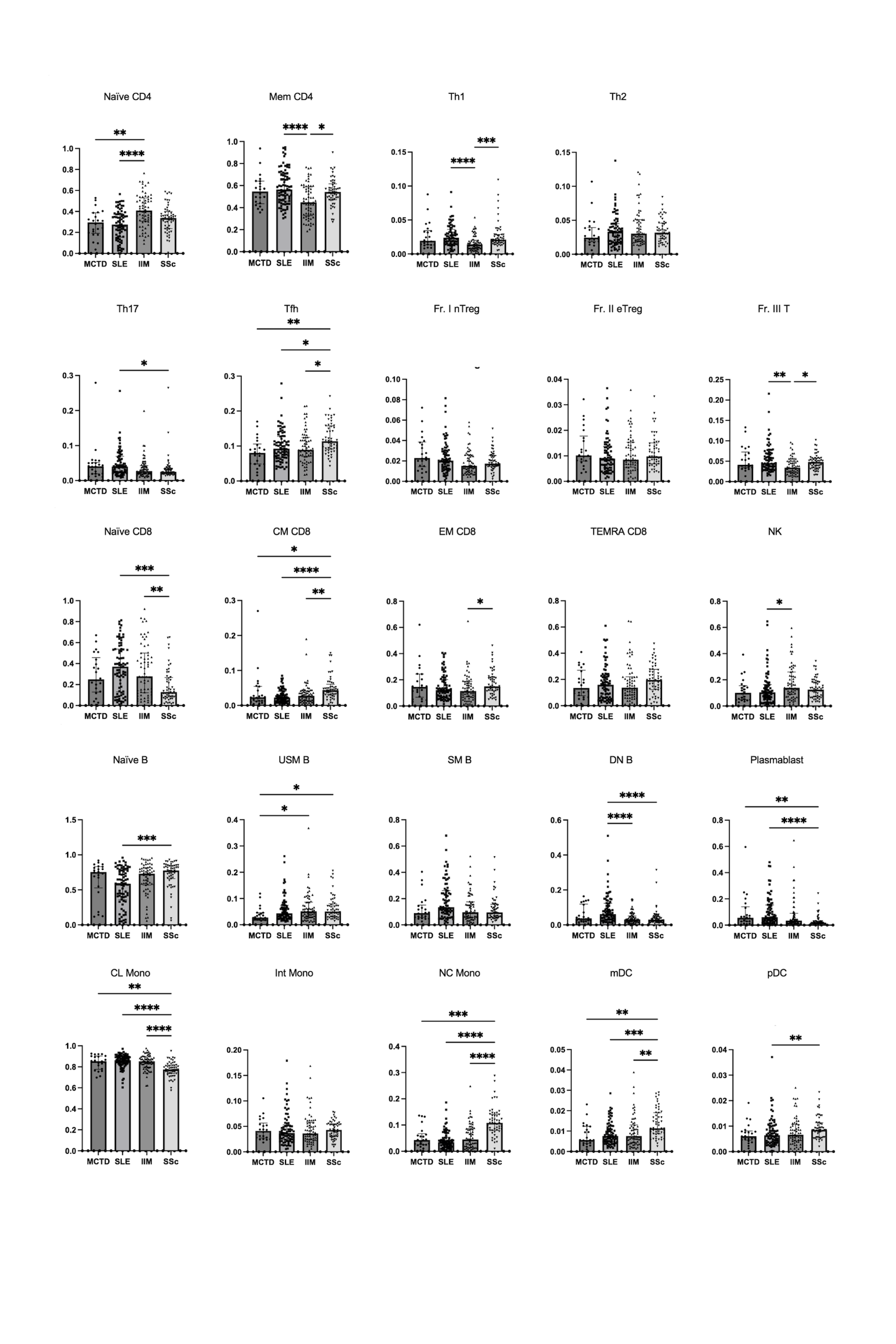


**Supplemental Figure S2.** **Volcano plot highlighting differentially expressed genes in TEMRA CD8 T cells.**


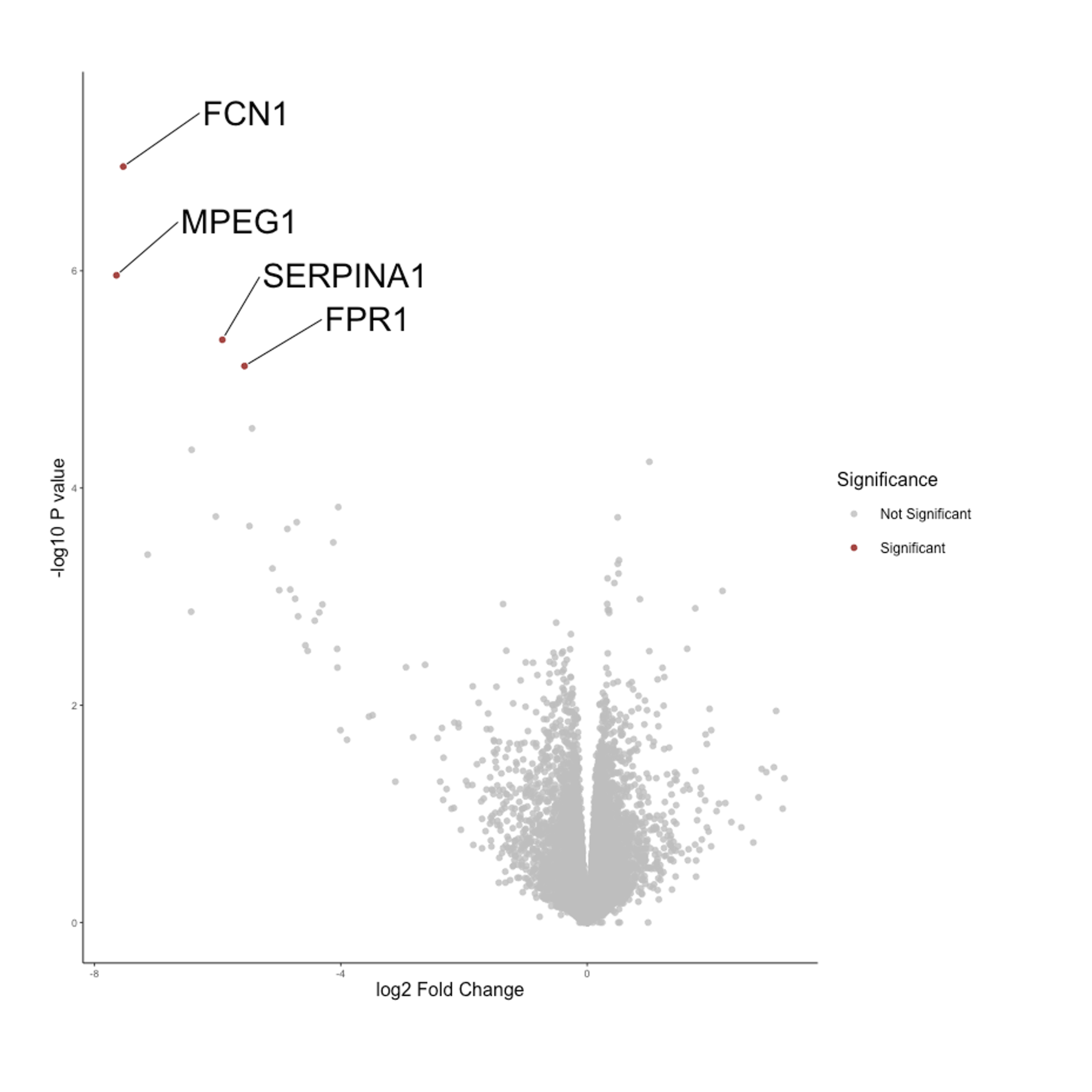

Supplement: keae158_Supplementary_Data [file keae158_supplementary_data.docx]
